# Evaluation of dietary inclusion of a blend of essential oils and probiotics for growing beef cattle

**DOI:** 10.1093/tas/txaf157

**Published:** 2025-11-25

**Authors:** Federico Podversich, Thiago L M Ribeiro, Forest L Francis, Warren C Rusche, Zachary K F Smith

**Affiliations:** Department of Animal Science, South Dakota State University, Brookings, SD 57007, United States; Department of Animal Science, South Dakota State University, Brookings, SD 57007, United States; Department of Animal Science, South Dakota State University, Brookings, SD 57007, United States; Department of Animal Science, South Dakota State University, Brookings, SD 57007, United States; Department of Animal Science, South Dakota State University, Brookings, SD 57007, United States

**Keywords:** *bacillus*, beef cattle, essential oils, feedlot, monensin

## Abstract

An experiment was conducted to compare the effects of feeding a proprietary blend of probiotics (*Bacillus licheniformis* and *Bacillus subtilis*) mixed with a blend of monoterpene and phenylpropene essential oils (EO), known as Palisade (Sulutions, Frankfort, KS) against monensin sodium, and their combination, on intake and growth performance responses of growing beef steers. Two hundred and forty Charolais × Angus crossbred steers (initial BW 340 ± 26.7 kg, ∼ 6 ± 1 month of age) were used. All steers received a basal corn-silage-based diet, with the inclusion of different feed additives: A) Palisade at 0.02% of the diet DM (PAL), B) Monensin sodium at 28 g/Ton (MON), and C) the combination of PAL and MON (PAL + MON). Steers were housed in 30 pens with 10 pens/treatment, and 8 steers/pen. Steers were fed for 83 days, and BW was measured every 28 days, and digestibility was determined from three fecal samplings using insoluble Ash as an internal marker. On d 83, steers fed MON were 8.9 and 11.8 kg heavier than steers fed the PAL or PAL + MON diets, respectively (*P = *0.01), with no difference between the last two. As a result, average daily gain (ADG) was greater for MON-fed steers, compared with steers fed with PAL or PAL + MON (*P = *0.01), without differences between the last two. Steers fed with the PAL + MON consumed less feed (*P = *0.01) than those fed either PAL or MON, with no difference between the last two. The cumulative gain to feed ratio (GF) was greater for MON-fed steers, compared with steers fed with PAL or PAL + MON (*P <*0.01), with no difference between the last two (*P >*0.10). Similarly, apparent Net energy of gain was greater for MON-fed steers than for PAL-fed steers (*P = *0.01), while PAL + MON steers were intermediate and did not differ with the other two groups. Around the days of nutrient digestibility determination, steers fed the PAL + MON diet consumed less (*P <*0.01) dry matter than steers receiving the PAL and MON diets, with no difference between the last two. No differences were observed in apparent total tract nutrient digestibility of nutrients (*P >*0.44), nor in the gross energy determined with a bomb calorimeter from feed and feces (*P >*0.76). Hence, the GE intake was reduced (*P <*0.01) for the PAL + MON-fed steers compared with steers fed with PAL or MON diets, with no difference between the last two. Steers fed with monensin alone had greater intake, body weight gain and feed efficiency than those fed PAL or PAL + MON.

## Teaser

The combination of Palisade with Monensin reduced the dry matter intake of growing steers. Growth performance was the best when feeding only monensin, compared with feeding Palisade or the combination of Palisade and Monensin.

## Introduction

The growing pressure on the use of natural alternatives to ionophores in beef cattle diets motivates the evaluation of alternative feed additives. Also, there is a potential benefit in the combination of these alternatives with monensin to impact both the rumen and the intestine. This combination is expected to aid in the control of systemic inflammation that, in turn, might reduce the need to use in-feed antimicrobials to control disease.

*Bacillus-based* direct-fed microbials (DFM) have been shown to reduce harmful pathogenic bacteria in the gastrointestinal tract. *Bacillus* species can modify the microbial population, alter the fermentation patterns, and improve nutrient digestibility. *Bacillus* spores are highly resilient and can survive in extreme environments and become active when conditions are more favorable. *Bacillus* spp. can grow both in the rumen and in the intestine and are characterized by producing several enzymes (Qiao et al. 2010; van Dijl and Hecker 2013; Fuerniss et al. 2022). Furthermore, the ruminal impact of *Bacillus*-based probiotics appears to be related to alterations in the existing microbiome as compared to colonization by the *Bacillus* itself (Schofield et al. 2018; Maderal 2022). In addition, this probiotic has demonstrated post-ruminal gastrointestinal tract effects, including immune stimulation, competitive pathogen exclusion, synthesis of antimicrobial substances, and alteration of the intestinal microbiome, among others (Elshaghabee et al. 2017; Maderal 2022). As expected, these effects can lead to alterations in the health and performance of cattle. While some of the previous studies indicate improved feed efficiency of cattle receiving Bacillus-based probiotics (Calaca et al. 2022; Dias et al. 2022; Lopez et al. 2024; Cordeiro et al. 2024), other studies failed to demonstrate an improvement in growth performance (Maderal 2022), with the reasons for this discrepancy remaining unclear.

Essential oils (EO) have been shown to reduce inflammation and modulate immune function. Terpenoids and phenylpropanoids interact with the bacterial cell membrane (Davidson and Naidu 2000; Dorman and Deans 2000; Ultee et al. 2002; Calsamiglia et al. 2007), with greater intensity on gram-positive bacteria (Calsamiglia et al. 2007). Some of the most common Monoterpenes used as feed additives are Thymol and Carvacrol, which are commonly found in plants such as Oregano and Thyme. These compounds are known to have potent antimicrobial activity (Calsamiglia et al. 2007). While not completely elucidated, Ultee et al. (2002) hypothesized an antimicrobial mechanism similar to ionophores, where Carvacrol alters the cytoplasmic ion balance. Their hypothesis involves an ion exchange between the cytoplasm and the external solution, where carvacrol enters the cell and releases H+ (becoming undissociated), while it captures K+, which is then transported to the outside of the cell (Ultee et al. 2002). Phenylpropanoids may have different modes of action but ultimately affect metabolic processes of the cell (Ultee et al. 2002). Some of the most used Phenylpropanoids as feed additives are cinnamaldehyde (from cinnamon), eugenol (from clove), and anethol (from anise, ­Calsamiglia et al. 2007). In the review by Calsamiglia et al. (2007), they imply that most essential oils and active components when evaluated at high doses can inhibit rumen microbial fermentation, which confirms the antimicrobial activity. In a dose-dependent manner, and with variation between different compounds, the EO were able to modify rumen fermentation by changing VFA production, protein metabolism, or both, reducing the acetate-to-propionate ratio, and increasing the butyrate concentration (Calsamiglia et al. 2007).

The objective of this study was to compare the effects of feeding a proprietary blend of probiotics (*Bacillus licheniformis* and *Bacillus subtilis*) mixed with a blend of monoterpene and phenylpropene essential oils (EO), known as Palisade (Sulutions, Frankfort, KS) against monensin sodium, and their combination, on intake and growth performance responses of growing beef steers. Our hypotheses were: 1) the blend of probiotics and essential oils will result in similar intake and growth performance as compared to monensin sodium, and 2) mixing the blend of probiotics and essential oils with monensin sodium will have a synergic effect on growth performance.

## Material and methods

### Institutional animal care and use approval

The experiment was conducted at the Ruminant Nutrition Center (RNC) in Brookings, SD between January 2024 and April 2024. The animal care and handling procedures used in this study were approved by the South Dakota State University Animal Care and Use Committee (Approval Number: 2412-015E).

### Treatment diets, animals and housing

All animals received a common corn-silage-based diet, with the inclusion of different feed additives: A) a proprietary blend of probiotics and EO (Palisade) at 0.02% of the diet DM (PAL), B) Monensin sodium at 28 g/Ton (MON), and C) the combination of PAL and MON (PAL + MON). A detailed description of the diets is presented in Table 1. The Palisade feed additive was previously mixed with approximately 25% of the DDGS fed in the diet, which served as a carrier for the PAL and PAL + MON diets. A medicated liquid supplement was used to deliver monensin to the MON and PAL + MON treatments, while a non-medicated liquid supplement was used for the PAL treatment. No tylosin phosphate was fed during the trial.

**Table 1.** Ingredients and nutrient composition of diets fed to beef steers.

	Treatments^a^		
	PAL	PAL + MON	MON
**Ingredients, % diet DM**			
** Corn silage**	64.11	64.10	64.11
** Dry-rolled corn**	10.30	10.30	10.31
** Dried Distillers’ grains (DDGS)**	16.07	15.82	20.43
** DDGS as carrier for PAL^b^**	4.37	4.63	…
** Non medicated liquid supplement^c^**	5.15	…	…
** Medicated liquid supplement^c^**	…	5.15	5.15
**Nutrient composition^d^**			
** DM, % of as fed**	46.7	46.7	46.7
** CP, % of DM**	13.0	13.0	13.0
** NDF, % of DM**	35.1	35.1	35.1
** ADF, % of DM**	17.7	17.7	17.7
** ASH, % of DM**	6.7	6.7	6.7
**Energy^e^**			
** NEm, Mcal/kg**	1.79	1.79	1.79
** NEg, Mcal/kg**	1.17	1.17	1.17

a For 84 days, steers received a corn silage-based diet, containing either a proprietary blend of probiotics (Bacillus licheniformis and Bacillus subtilis) and essential oils (Palisade, Sulutions, Frankfort, KS) at 0.02% of the diet DM (PAL), monensin sodium at 28 g/Ton (MON), or C) the combination of PAL and MON (PAL + MON).

b The DDGS carrier contained 0.44% of a blend of (Palisade, Sulutions, Frankfort, KS), for a final concentration in the diet DM of 0.02%.

c Prepared by Midwest PMS LLC, to fulfill the requirements for vitamins and minerals (NASEM 2016). The medicated liquid supplement was formulated so that the final diet contains 28 g/TON of monensin (Rumensin-90, Elanco Animal Health).

d DM: dry matter, CP: crude protein, NDF: neutral detergent fiber, ADF: acid detergent fiber.

e NEm: Net Energy for maintenance, Neg: Net Energy for gain.

The experiment used two hundred and forty Charolais × Angus crossbred steers (initial BW 340 ± 26.7 kg, ∼6 ± 1 month of age) that were weaned in Mid-October of 2023. The steers were housed in 7.62 × 7.62 m concrete surface pens with 7.62 m of linear bunk-space at the RNC. Steers were distributed in 10 blocks based on pen location within the feedyard, with BW being balanced among blocks. Pens contained 8 steers (*n* = 80 steers/treatment), each treatment had ten replicate pens, and the study was conducted as a randomized complete block design. The steers were acquired from a single-source in Western South Dakota and transported 319 miles (6.5 hours transit) to the Ruminant Nutrition Center (RNC) in Brookings, SD in mid-October of 2023 and were used in a receiving phase study. Upon arrival in October, all steers were identified using a unique identification ear tag, then vaccinated against viral respiratory diseases (Bovishield Gold ONE SHOT, Zoetis) and clostridial species (Ultrabac 7/Somubac, Zoetis), and administered pour on moxidectin (Cydectin, Elanco) according to label directions. Receiving phase treatment (*n* = 2 treatments) was equally balanced across the three experiment treatments during the backgrounding phase study.

The morning prior to study initiation, all steers were subjected to individual BW determination that was used for allotment purposes, and were treated for external parasites (Cylence, Elanco). The following morning, steers were re-weighed, and test diets were initiated. The initial BW used was the average of the two body weights collected over two consecutive days.

### Management

Throughout the entire study, steers were fed 2X daily in a 50:50 split. Bunks were managed using a slick bunk management approach such that bunks were devoid of feed by 0800 h most mornings. Feed was manufactured in a stationary mixer (2.35 m^3^; Roto-Mix, Dodge City, KS), all ingredients were added into the mixer to the nearest pound, and feed was delivered to each pen separately (weighed out of the stationary mixer to the nearest pound) into a feed delivery wagon that is not mounted on load cells. Batching sequence was as follows: PAL (5 pens), PAL (5 pens), PAL + MON (5 pens), PAL + MON (5 pens), MON (5 pens), and finally MON (5 pens). Following each batch of feed, long stem grass hay (∼8 lbs) was added to the mixer and used to flush out all residual feed remaining in the mixer.

All steers were individually weighed approximately every 28 d throughout the experimental period for the calculation of growth performance responses. Ingredient samples were collected weekly for DM determination and TMR samples were collected weekly and composited monthly for nutrient determination.

### Growth performance

Steers were individually weighed on approximately d −1, 1, 28, 56 and 84. Growth performance was calculated on a deads and removals excluded basis. Steers were weighed in a hydraulic squeeze chute mounted on top of load cells (scale readability ±0.91 kg). All BW data were shrunk 4% to account for gastrointestinal tract fill. Average daily gain (ADG) was calculated as the difference between initial and final shrunk BW, divided by the days on feed, for each interim period. Feed conversion efficiency (G:F) was calculated from ADG/DMI.

### Dietary net energy utilization calculations

Growth performance was used to calculate performance-based dietary NE in order to determine efficiency of dietary NE utilization. The performance-based dietary NE was calculated from daily energy gain (EG; Mcal/d): EG = ADG^1.097^ × 0.0557 W^0.75^, where W is the mean equivalent shrunk BW [kg; (NASEM 2016)] from median feeding shrunk BW and final BW at 28%. Adjusted final BW at 28% empty body fat (AFBW) was assumed to be 625 kg from previous studies conducted by our group with similar cattle (Smith 2020). Equivalent shrunk body weight was calculated as: (median feeding shrunk BW × [478/625], kg) from NASEM (2016). Maintenance energy (EM) were calculated by the equation: EM = 0.077 × median feeding shrunk BW^0.75^. Dry matter intake is related to energy requirements and dietary NEm (Mcal/kg) according to the following equation: DMI = EG/(0.877NEm − 0.41), and can be resolved for estimation of dietary NEm by means of the quadratic formula $x=\frac{-b\pm \sqrt{b^{2}-4ac}}{2c}$, where *a* = −0.41EM, *b* = 0.877EM + 0.41DMI + EG, and *c* = −0.877DMI (Zinn and Shen 1998). Dietary NEg was derived from NEm using the following equation: NEg = 0.877NEm − 0.41 (Zinn and Shen 1998). Observed-to-expected (O:E) NEm and NEg was calculated from observed dietary NE values for maintenance or gain divided by tabular estimates of NE for maintenance or gain (Preston 2017).

### Sample analysis

Actual diet formulations (Table 1) were based upon weekly DM analysis (drying at 60°C until no weight change was observed) and corresponding feed batching records. After weekly DM (method no. 935.29; AOAC 2012), proximate analysis of each ingredient (except for liquid supplement) was conducted using weekly samples composited on a monthly basis, and analyzed for N (method no. 968.06; AOAC 2016; Rapid Max N Exceed; Elementar; Mt. Laurel, NJ, USA), where crude protein (CP) was determined from *N* × 6.25, and ash (method no. 942.05; AOAC 2012). Percentages of neutral detergent fiber (NDF) acid detergent fiber (ADF) of corn grain were assumed to be 9.7% and 3% (NASEM 2016). Analysis of NDF and ADF composition for all other ingredients was conducted as described by Goering and VanSoest (1970) using heat-stable α-amylase and sodium sulfite as described by Van Soest et al. (1991) in an Ankom 200 Fiber Analyzer (Ankom Technology Corp.). Tabular energy values according to Preston (2017) were utilized for the Net energy of all ingredients.

*Digestibility estimates.* Estimation of the apparent total tract digestibility (ATTD) of nutrients was conducted based on Beck et al. (2023). Fecal samples were collected via rectal palpation on days 28, 56, and 84 and composited by pen, placed on ice and then frozen at −20°C for later analysis. Diet samples were collected for the three days prior to fecal sampling to determine nutrient intake. Dry matter, organic matter (OM; DM less ash), NDF, and starch for both diet and fecal samples were analyzed using the same procedures as described for ingredients analysis. Insoluble Ash was utilized as an internal marker. Nutrient digestibility determinations from the 3 sampling days were averaged by pen prior to statistical analysis. The ATTD of DM, OM, NDF, and Starch was calculated using the following formula:


$$\mathrm{ATTD}=\;100-100\times \;[\left(\frac{\mathrm{marker}\;\mathrm{concentration}\;\mathrm{in}\;\mathrm{feed}}{\mathrm{marker}\;\mathrm{concentration}\;\mathrm{in}\;\mathrm{feces}})\times \;(\frac{\mathrm{nutrient}\;\mathrm{concentration}\;\mathrm{in}\;\mathrm{feces}}{\mathrm{nutrient}\;\mathrm{concentration}\;\mathrm{in}\;\mathrm{feed}}\right)]$$

*Dietary digestible energy determination.* The gross energy (GE) of feed and feces was obtained in feed using a bomb calorimeter (Parr 6400 calorimeter, Parr Instruments Co., Moline, IL). Diet and feces samples were ground through a 1 mm screen. One gram of ground sample was pressed into a pellet using a pellet press (Parr Instruments Co., Moline, IL) and weight into a crucible to be analyzed by the bomb calorimeter, according to standard protocol (AOAC 1990). All samples were analyzed in duplicate and re analyzed if the difference was more than 5%. Energy determinations from the 3 sampling days were averaged by pen prior to statistical analysis.

### Weather monitoring

Climatic variables were obtained every 30 minutes from a weather station located near the RNC center prior to and throughout the experimental period (January to April of 2024).

### Statistical analysis

Data were analyzed as a randomized complete block design using the GLIMMIX procedure of SAS 9.4 (SAS Inst. Inc., Cary, NC) with the pen as the experimental unit. The model included the fixed effects of dietary treatment and block. Within the feedyard, three adjacent cattle pens formed the block, with one pen per treatment in each block. Differences among treatments were declared at *P ≤*0.05, and tendencies were considered when 0.10 > *P >*0.05. Tukey’s adjustment was used to conduct means separation when the *P-value* for treatment was significant.

## Results

Growth performance data is presented in Table 2. As per design, no differences were observed for initial BW among treatments (*P *= 0.25). On d 28, no differences were observed among treatments on BW (*P *= 0.22), and neither for ADG, DMI, and GF ratio (*P > *0.21).

**Table 2.** Growth performance of beef steers.

	Treatment^1^				
Item	PAL	PAL + MON	MON	SEM^2^	*P-*value^3^
**Pens, n**	10	10	10	…	…
**Steers, n**	80	80	80	…	…
**Body weight, kg**					
** Initial**	326.6	327.5	327.0	1.27	0.25
** d 28**	364.2	367.4	367.0	1.68	0.22
** d 56**	399.8^b^	394.5^b^	408.8^a^	1.68	0.01
** d 83**	437.8^b^	434.9^b^	446.7^a^	1.82	0.01
**Average daily gain, kg**					
** Initial to d 28**	1.35	1.42	1.43	0.045	0.42
** d 28 to d 56**	1.26	0.97	1.49	0.048	0.09
** d 56 to d 83**	1.41	1.49	1.41	0.034	0.15
** Initial to d 83**	1.34^b^	1.29^b^	1.44^a^	0.023	0.01
**Dry matter intake, kg**					
** d 0 to d 28**	8.87	8.83	8.74	0.600	0.32
** d 28 to d 56**	9.13^b^	8.23^c^	9.50^a^	0.098	<0.01
** d 56 to 83**	10.41^a^	10.00^b^	10.23^ab^	0.105	0.04
** Initial to d 83**	9.46^a^	9.01^b^	9.48^a^	0.071	0.01
**Gain to Feed, kg/kg**					
** Initial to d 28**	0.152	0.161	0.163	0.0046	0.21
** d 28 to d 56**	0.138^b^	0.118^c^	0.157^a^	0.0051	<0.01
** d 56 to 83**	0.135^b^	0.149^a^	0.137^b^	0.0029	<0.01
** Initial to d 83**	0.142^b^	0.144^b^	0.152^a^	0.0019	0.01
**Observed net energy (NE), Mcal/kg^4^**					
** Maintenance**	1.67^b^	1.70^ab^	1.74^a^	0.013	0.01
** Gain**	1.05^b^	1.08^ab^	1.12^a^	0.012	0.01
**Observed-to expected NE^5^**					
** Maintenance**	0.93^b^	0.95^ab^	0.97^a^	0.008	0.01
** Gain**	0.90^b^	0.92^ab^	0.95^a^	0.009	0.01

1 For 84 days, steers received a corn silage-based diet (1.17 Mcal/kg NEg), containing either a proprietary blend of probiotics (*Bacillus licheniformis* and *Bacillus subtilis*) and essential oils (Palisade, Sulutions, Frankfort, KS) at 0.02% of the diet DM (PAL), monensin sodium at 28 g/Ton (MON), or C) the combination of PAL and MON (PAL + MON).

2 Standard Error of the mean (*n* = 10 pens/treatment).

3 Observed significance for the effect of treatment.

4 Net Energy for maintenance and gain based on performance were calculated based on Zinn et al. (2008).

5 For the observed over expected NE, expected NE was calculated using tabular data (NASEM 2016).

a, b, c Within a row, means with different superscripts differ, *P ≤ *0.05.

On day 56, MON-fed steers were 8.9 and 14.2 kg heavier than steers receiving the PAL or PAL + MON diets (*P *= 0.01), respectively, with no differences observed between the last two. Between d 28 to 56 of the trial, a tendency was observed for ADG (*P = *0.09), while a difference was observed for DMI (*P < *0.01), where MON-fed steers had the greatest intake, followed by PAL-fed steers, and with steers receiving the PAL + MON having the least DMI. Similarly, GF ratio differed among treatments (*P < *0.01), with MON-fed steers having the greatest GF, followed by PAL-fed steers, and with steers receiving the PAL + MON exhibiting the lowest GF.

On day 83, MON-fed steers were 8.9 and 11.8 kg heavier than steers fed the PAL or PAL + MON diets, respectively (*P *= 0.01), and no difference was observed between the last two. Between d 56 and 83 of the trial, no difference was observed for ADG (*P *= 0.15), while a difference was observed for DMI (*P *= 0.01), where PAL-fed steers ate more than PAL + MON-fed steers, while MON-fed steers were intermediate and did not differ with the other two groups. A difference was observed for GF ratio (*P < *0.01), with PAL + MON fed steers having greater GF ratio than MON-fed and PAL-fed steers, with the last two not differing between them.

Cumulatively, from the beginning of the trial until the end on day 83, the MON-fed steers had greater ADG than steers fed with PAL or PAL + MON (*P *= 0.01), with the last two not differing between them. No difference was observed on DMI between MON-fed and PAL-fed steers (*P > *0.10), but both groups had greater DMI than PAL + MON-fed steers (*P   *0.01). The MON-fed steers had a greater GF ratio than steers fed with PAL or PAL + MON (*P *= 0.01), with the last two not differing between them.

Performance calculated NEm and NEg was greater for MON-fed steers than for PAL-fed steers (*P *= 0.01), while PAL + MON steers were intermediate and did not differ with the other two treatment groups. Similarly, the ratio of observed over expected NEm and NEg was greater for MON-fed steers than for PAL-fed steers (*P *= 0.01), while PAL + MON steers were intermediate and did not differ with the other two treatment groups.

Apparent total tract digestibility data is presented in Table 3. Around the days of nutrient digestibility determination, steers receiving the PAL + MON diet consumed less (*P <*0.01) dry matter than steers receiving the PAL and MON diets, with these last two not differing between them. No differences were observed in apparent total tract nutrient digestibility for any of the nutrients evaluated (*P > *0.44). Gross energy determined with bomb calorimeter from feed and feces did not differ among treatments (*P > *0.76). As a result of the difference on intake, the GE intake was reduced (*P < *0.01) for the steers receiving the PAL + MON diet compared with steers receiving the PAL or MON diets, with these last two not differing between them. Also, fecal GE output and calculated DE intake tended to be lower for the PAL + MON group (*P < *0.07), compared with the other two groups. Finally, dietary DE and ME did not differ among treatments (*P *= 0.70).

**Table 3.** Apparent total tract digestibility and digestible energy determinations.

	Treatment^1^				
Item	PAL	PAL + MON	MON	SEM^2^	*P-*value^3^
**Dry matter intake, kg/d**	9.03^a^	8.18^b^	9.41^a^	0.124	<0.01
**Fecal output, kg/d**	3.53	3.24	3.87	0.186	0.08
**Digestibility^4^, % of intake**					
** Dry matter (DM)**	61.1	60.4	58.7	2.00	0.70
** Organic matter**	63.2	62.6	60.8	1.93	0.67
** Neutral detergent fiber**	41.2	39.9	37.8	2.5	0.64
** Starch**	89.0	88.5	87.1	1.06	0.44
**Energetics^5^**					
** Diet Gross Energy (GE), Mcal/kg of DM**	4.21	4.22	4.21	0.010	0.73
** Feces GE, Mcal/kg of DM**	4.08	4.10	4.08	0.017	0.76
** GE intake, Mcal/d**	38.0^a^	34.5^b^	39.6^a^	0.50	<0.01
** Fecal GE Output, Mcal/d**	14.4	13.2	15.8	0.72	0.07
** Digestible Energy (DE) intake, Mcal/d**	23.6	21.3	23.8	0.79	0.06
** DE, Mcal/kg of DM**	2.62	2.60	2.53	0.083	0.70
** Metabolizable energy, Mcal/kg of DM**	2.23	2.21	2.14	0.083	0.70

1 For 84 days, steers received a corn silage-based diet (1.17 Mcal/kg NEg), containing either a proprietary blend of probiotics and essential oils (Palisade, Sulutions, Frankfort, KS) at 0.02% of the diet DM (PAL), monensin sodium at 28 g/Ton (MON), or C) the combination of PAL and MON (PAL + MON).

2 Standard Error of the mean (*n* = 10 pens/treatment).

3 Observed significance for the effect of treatment.

4 Determined using insoluble ash as internal marker.

5 Determined using Isoperibol bomb calorimetry (Parr 1261 Isoperibol Bomb Calorimeter, Parr Instrument Co., Moline, IL, USA).

a, b, c Within a row, means with different superscripts differ, *P* *≤* 0.05.

## Discussion

**Fig. 1.**
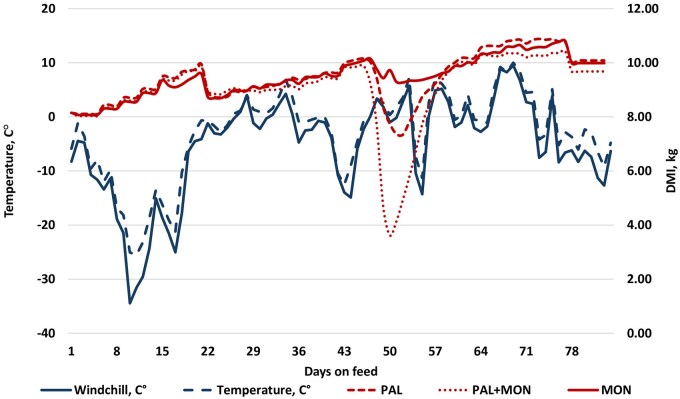
Temperature and windchill, and feed intake of beef steers fed a growing diet, along the days on feed (4 January to 28 March of 2024). For 84 days, steers received a corn silage-based diet (1.17 mcal/kg NEg), containing either a proprietary blend of probiotics and essential oils (Palisade, Sulutions, Frankfort, KS) at 0.02% of the diet DM (PAL), monensin sodium at 28 g/ton (MON), or C) the combination of PAL and MON (PAL + MON).

A major weather event occurred prior to the 56-d intermediate weigh day characterized by rapid changes in temperature and windchill (Fig. 1). Three pens on the PAL + MON treatment dropped in intake with the weather change (individual pen data not shown). Cattle recovered in intake in the succeeding period. None of the cattle in those pens exhibited ill health symptoms and no treatments were administered. Whether reduced intake was caused by the weather event, the treatment or the interaction between weather event and treatment is not clear.

Similarly to our study, Cordeiro et al. (2024) observed a reduction in feed intake by 0.4 kg/d when a *Bacillus* blend was included in the diet of finishing Nellore bulls. Yet, in the mentioned study, the reduction on intake resulted in improved GF ratio of the finishing bulls (Cordeiro et al. 2024). Silva et al. (2024) conducted a study with ruminal cannulated steers fed a finishing diet containing 50% Starch and 20% NDF. These authors found that the dietary inclusion of a blend of *Bacillus* resulted in a slower rate of ruminal in situ organic matter degradation (Silva et al. 2024). Yet, in the same study, intake and apparent total tract digestibility of organic matter were unaffected by treatment, while ruminal pH, ruminal ammonia nitrogen (NH_3_N), and total tract ADF digestibility were increased by *Bacillus* inclusion in the diet (Silva et al. 2024). Such findings suggest that *Bacillus* had a modulatory effect on the ruminal microbiome, leading to a reduced starch degradation in the rumen, with improved pH stabilization, increased ADF degradation and ruminal NH_3_N accumulation. In agreement, Maderal (2022), observed a reduction in apparent total tract digestibility of starch with the dietary inclusion of a *Bacillus* spp. blend on beef heifers consuming a low energy grower diet based on sorghum-silage. In the same study, supplementation with *Bacillus* spp. did not influence growth performance of growing beef heifers.

The combination of monensin with the *bacillus* and essential oils blend may have had a major impact on the ruminal microbiome, diminishing ruminal degradation of feeds to such an extent with negative consequences, and leading to a reduction in feed intake. Yet, with the current information obtained from this experiment, this hypothesis cannot be demonstrated. Feeding only monensin resulted in 5% greater feed conversion overall, as compared with combining monensin with the *Bacillus* and essential oils blend. In the same line, when feeding only the *Bacillus* and essential oils blend, the feed conversion of the steers was 6.6% less compared with feeding Monensin. Because of the lack of negative control, without a feed additive, we cannot distinguish if the *Bacillus* and essential oils blend alone reduced growth performance or if it just did not improve it, as we expect monensin to do. Yet, we were able to determine that the combination of monensin with *bacillus* and essential oils affected negatively intake and growth performance of the steers, as compared with feeding monensin alone.

This experiment was not designed to fully explore the effects of Palisade on nutrient digestibility; therefore, these results should be interpreted with caution. Yet, the study by Beck et al. (2023) determined a Pearson correlation of 0.87 for apparent total tract digestibility of DM, between the estimates for total collection and two-time sampling using insoluble Ash as a marker. The findings from this study do not indicate a relevant effect of the Palisades product on apparent total tract nutrient digestibility as compared with Monensin.

## Conclusions

In the context of this study, feeding monensin alone resulted in greater intake, body weight gain and feed efficiency than feeding Palisade or the combination of Palisade with monensin. The erratic weather conditions during this trial make the interpretation of results complex. Further research is needed to clarify the mechanisms behind the observed responses.

## Data Availability

Data can be made available upon reasonable request to Z.K.S.

## LIST OF ABBREVIATIONS:

ADF: acid detergent fiber
ADG: average daily gain
AFBW: estimated body weight at 28% empty body fat
BW: body weight
CP: crude protein
DM: dry matter
DMI: dry matter intake
DOF: days on feed
DRC: dry rolled corn grain
EBF: empty body fat
EBW: empty body weight
EG: daily energy gain
EM: maintenance energy required
F:G: feed to gain
G:F: gain to feed ratio
HCW: hot carcass weight
MBW: metabolic body weight
MDGS: modified distiller’s grains with solubles
NDF: neutral detergent fiber
NE: net energy
NEg: net energy of gain
NEm: net energy of maintenance
O:E: observed-to-expected
OM: organic matter
RCBD: randomized complete block design
RFID: radio-frequency identification
SBW: shrunk body weight
YG: yield grade

## References

[txaf157-B1] AOAC. 2012. Official methods of analysis. 19th ed. Assoc. Off. Anal. Chem., Arlington, VA.

[txaf157-B2] AOAC. 2016. Official methods of analysis of AOAC International. 20th ed. Assoc. Off. Anal. Chem., Arlington, VA.

[txaf157-B3] Beck M. R. et al. 2023. Assessing different sampling regimens for estimating dietary characteristics using internal markers. Appl. Anim. Sci. 39:411–422. 10.15232/aas.2023-02452.

[txaf157-B4] Calaca A. M. M. et al. 2022. Effect of a Bacillus probiotic strain on Nellore cattle finished on pasture during the dry season. Livest. Sci. 264:105068. 10.1016/jlivsci.2022.105068

[txaf157-B5] Calsamiglia S. , Busquet M., Cardozo P. W., Castillejos L., Ferret A. 2007. Invited review: essential oils as modifiers of rumen microbial fermentation. J. Dairy Sci. 90:2580–2595. 10.3168/jds.2006-644. 17517698

[txaf157-B6] Cordeiro M. W. S. , Cappellozza B. I., de Melo N. N., Bernardes T. F. 2024. Effects of a *bacillus*-based direct-fed microbial on performance, blood parameters, fecal characteristics, rumen morphometrics, and intestinal gene expression in finishing beef bulls. J. Anim. Sci. 102:skae259. 10.1093/jas/skae259 39248595 PMC11439149

[txaf157-B7] Davidson P. M. , Naidu A. S. 2000. Phyto-phenols. In: Naidu A. S., editor, Natural food antimicrobial systems. CRC Press, Boca Raton, FL, p. 265–293.

[txaf157-B8] Dias B. G. C. et al. 2022. Effects of feeding different probiotic types on metabolic, performance, and carcass responses of *Bos indicus* feedlot cattle offered a high-concentrate diet. J. Anim. Sci. 100:skac289. 10.1093/jas/skac289 36055763 PMC9584148

[txaf157-B9] Dorman H. J. D , Deans S. G. 2000. Antimicrobial agents from plants: antibacterial activity of plant volatile oils. J. Appl. Microbiol. 88:308–316. 10736000 10.1046/j.1365-2672.2000.00969.x

[txaf157-B10] Elshaghabee F. M. F. , Rokana N., Gulhane R. D., Sharma C., Panwar H. 2017. Bacillus as potential probiotics: Status, concerns, and future perspectives. Front. Microbiol. 8:1490. 10.3389/fmicb.2017.01490 28848511 PMC5554123

[txaf157-B11] Fuerniss L. K. , Kreikemeier K. K., Reed L. D., Cravey M. D., Johnson B. J. 2022. Cecal microbiota of feedlot cattle fed a four-species *bacillus* supplement. J. Anim. Sci. 100:skac258. 10.1093/jas/skac258 35953238 PMC9576023

[txaf157-B12] Goering H. K. , VanSoest P. J. 1970. Forage fiber analyses (apparatus, reagents, procedures, and some applications). Agric. Handbook No. 379. ARS, USDA.

[txaf157-B14] Lopez A. M. , Sarturi J. O., Johnson B. J., Woerner D. R., Henry D. D., Ciriaco F. M., Silva K. G. S., Rush C. J. 2024. Effects of bacterial direct-fed microbial combinations on beef cattle growth performance, feeding behavior, nutrient digestibility, ruminal morphology, and carcass characteristics. J. Anim. Sci. 102: skae004. 10.1093/jas/skae004 38190444 PMC10836501

[txaf157-B15] Maderal A. 2022. Bacillus Spp. as a probiotic to enhance beef cattle production [master’s thesis]. University of Florida. https://ufdc.ufl.edu/ufe0059570/00001

[txaf157-B17] NASEM. 2016. Nutrient requirements of beef cattle. 8th rev. ed. Natl. Acad. Press, Washington, DC. 10.17226/19014

[txaf157-B18] Preston R. L. 2017. Feed composition table. https://www.beefmagazine.com/cattle-nutrition/feed-composition-tables-how-to-use-the-2017-data-to-mix-the-best-feed-for-your-cattle

[txaf157-B19] Qiao G. H. , Shan A. S., Ma N., Ma Q. Q., Sun Z. W. 2010. Effect of supplemental *bacillus* cultures on rumen fermentation and milk yield in Chinese Holstein cows. J. Anim. Physiol. Anim. Nutr. (Berl). 94:429–436. 10.1111/j.1439-0396.2009.00926.x 19663976

[txaf157-B20] Schofield B. J. et al. 2018. Beneficial changes in rumen bacterial community profile in sheep and dairy calves as a result of feeding the probiotic *Bacillus amyloliquefaciens* H57. J. Appl. Microbiol. 124:855–866. 10.1111/jam.13688 29314469

[txaf157-B21] Silva K. G. S. et al. 2024. Effects of bacterial direct-fed microbial mixtures offered to beef cattle consuming finishing diets on intake, nutrient digestibility, feeding behavior, and ruminal kinetics/fermentation profile. J. Anim. Sci. 102:1–15. 10.1093/jas/skae003 PMC10833447 38183669

[txaf157-B22] Smith Z. K. 2020. Nose color of charolais × british crossbred beef steers alters body weight at a common degree of fatness and marbling score in steers reared under similar management from birth through finishing. Am. J. Anim. Vet. Sci. 15:252–256. 10.3844/ajavsp.2020.252.256

[txaf157-B23] Ultee A. , Bennik M. H. J., Moezelaar R. 2002. The phenolic hydroxyl group of carvacrol is essential for action against the food-borne pathogen *Bacillus cereus*. Appl. Environ. Microbiol. 68:1561–1568. 11916669 10.1128/AEM.68.4.1561-1568.2002 PMC123826

[txaf157-B24] van Dijl J. M , Hecker M. 2013. *Bacillus subtilis*: from soil bacterium to super-secreting cell factory. Microb. Cell Fact. 12:3–6. 10.1186/1475-2859-12-3 23311580 PMC3564730

[txaf157-B151] Van Soest P.J. , Robertson J.B., Lewis B.A. 1991. Methods for Dietary Fiber, Neutral Detergent Fiber, and Nonstarch Polysaccharides in Relation to Animal Nutrition. J. Dairy Sci. 74:3583–3597. 1660498 10.3168/jds.S0022-0302(91)78551-2

[txaf157-B25] Zinn R. A , Shen Y. 1998. An evaluation of ruminally degradable intake protein and metabolizable amino acid requirements of feedlot calves. J. Anim. Sci. 76:1280–1289. 10.2527/1998.7651280x 9621934

[txaf157-B26] Zinn R. A. , Barreras A., Owens F. N., Plascencia A. 2008. Performance by feedlot steers and heifers: daily gain, mature body weight, dry matter intake, and dietary energetics. J. Anim. Sci. 86:2680–2689. 10.2527/jas.2007-0561 18539825

